# Neurological and psychiatric safety of tafenoquine in *Plasmodium vivax* relapse prevention: a review

**DOI:** 10.1186/s12936-020-03184-x

**Published:** 2020-03-14

**Authors:** Stephan Duparc, Stephan Chalon, Scott Miller, Naomi Richardson, Stephen Toovey

**Affiliations:** 1 Medicines for Malaria Venture, Route de Pré-Bois 20, 1215 Geneva 15, Switzerland; 2 Bill and Melinda Gates Foundation, Seattle, USA; 3 Magenta Communications Ltd, Abingdon, UK; 4 Pegasus Research, London, UK

**Keywords:** Malaria, Tafenoquine, Neuropsychiatric, Adverse events, Anti-malarial

## Abstract

**Background:**

Tafenoquine is an 8-aminoquinoline anti-malarial drug recently approved as a single-dose (300 mg) therapy for *Plasmodium vivax* relapse prevention, when co-administered with 3-days of chloroquine or other blood schizonticide. Tafenoquine 200 mg weekly after a loading dose is also approved as travellers’ prophylaxis. The development of tafenoquine has been conducted over many years, using various dosing regimens in diverse populations.

**Methods:**

This review brings together all the preclinical and clinical data concerning tafenoquine central nervous system safety. Data were assembled from published sources. The risk of neuropsychiatric adverse events (NPAEs) with single-dose tafenoquine (300 mg) in combination with chloroquine to achieve *P. vivax* relapse prevention is particularly examined.

**Results:**

There was no evidence of neurotoxicity with tafenoquine in preclinical animal models. In clinical studies in *P. vivax* relapse prevention, nervous system adverse events, mainly headache and dizziness, occurred in 11.4% (36/317) of patients with tafenoquine (300 mg)/chloroquine versus 10.2% (19/187) with placebo/chloroquine; and in 15.5% (75/483) of patients with tafenoquine/chloroquine versus 13.3% (35/264) with primaquine (15 mg/day for 14 days)/chloroquine. Psychiatric adverse events, mainly insomnia, occurred in 3.8% (12/317) of patients with tafenoquine/chloroquine versus 2.7% (5/187) with placebo/chloroquine; and in 2.9% (14/483) of patients with tafenoquine/chloroquine versus 3.4% (9/264) for primaquine/chloroquine. There were no serious or severe NPAEs observed with tafenoquine (300 mg)/chloroquine in these studies.

**Conclusions:**

The risk:benefit of single-dose tafenoquine/chloroquine in *P. vivax* relapse prevention is favourable in the presence of malaria, with a low risk of NPAEs, similar to that seen with chloroquine alone or primaquine/chloroquine.

## Background

In a chemoprophylaxis setting, a number of anti-malarial drugs have been associated with neurological or psychiatric adverse events (NPAEs), in particular, the quinoline-methanol derivative mefloquine [1–4]. Although the mechanisms of such neurotoxicity are not always well understood or clearly defined, a considerable body of evidence has accumulated on this topic [5]. Despite this, it can be difficult to translate pre-clinical neurotoxicity findings, generally observed at toxicological doses, into the potential for clinical neurological or psychiatric reactions in malaria patients or those receiving malaria chemoprophylaxis. Additionally, NPAEs may be readily confounded by neurological or psychiatric signs and symptoms of acute malaria and underlying risk factors for psychiatric conditions.

The 8-aminoquinoline, tafenoquine, has been approved as monotherapy for travellers’ prophylaxis, with an approved dose of 200 mg/day for 3 days followed by a 200 mg weekly maintenance doses thereafter [6, 7]. Tafenoquine is the only once-weekly prophylaxis that can be used in areas with chloroquine- and mefloquine-resistant parasites. Tafenoquine can also provide an alternative weekly prophylaxis regimen to mefloquine, but potentially without the concerns regarding NPAEs that restrict mefloquine use [4, 8]. Thus, the NPAE profile of tafenoquine is of great clinical interest.

Tafenoquine has also been approved for *Plasmodium vivax* relapse prevention as a single-dose therapy (300 mg) in combination with the standard adult dose of chloroquine (1500 mg free base in staggered dosing over 3 days) [9–11]. Previously, the recommended treatment for *P. vivax* relapse prevention required 3-days of chloroquine plus 14-days of primaquine 15 mg or 30 mg once daily. Primaquine is also an 8-aminoquinoline, but adherence to the dosing schedule is poor, and the clinical effectiveness of unsupervised primaquine is similar to that of placebo [12, 13]. In contrast, single-dose tafenoquine offers the possibility of complete adherence and consistent clinical effectiveness [9–11]. Preventing clinical relapses associated with the re-activation of the dormant *P. vivax* hypnozoites would potentially have a significant impact on reducing the burden of malaria and accelerating *P. vivax* elimination in endemic countries. This review considers the available preclinical and clinical safety data to assess the potential for tafenoquine to induce NPAEs in the context of findings for other anti-malarial drugs.

## CNS adverse events of marketed anti-malarial drugs

### Preclinical evaluations

Neurotoxicity is a complex area for preclinical investigation. In vitro studies of cytotoxicity against human neuroblastoma cells or similar models are suggestive of neurotoxic potential, but the relevance of such results depends on drug penetration into the CNS, as well as pharmacokinetic and other factors, for example, metabolic status. In animal models, cytotoxicity and morphological damage are obvious outcomes, but may be restricted to very specific brain areas. Neurological effects can also derive from purely functional disruption, with no morphological damage, for example, convulsions induced by fluoroquinolones [14–16]. Thus, in modern drug development, neurobehavioural testing is used to detect any functional effects of drugs. This uses both observational and more complex methods, and multiple tests must be used to objectively evaluate different potential functional and behavioural impacts of the drug.

For older anti-malarial drugs, comprehensive neurotoxicological preclinical testing may not have been conducted. However, for new agents, supra-therapeutic or lethal drug doses are administered in animal models to attempt to induce observable lesions in CNS tissues. If lethal doses do not induce neurotoxicity, then the toxicities that caused death are deemed dose limiting. In such circumstances, a “safety factor” (typically > 10-fold) is applied to the pharmacokinetic exposures in animals in order to predict the “highest achievable exposure” for humans. Consequently, the absence of neurotoxicity findings in pre-clinical animal models at supra-therapeutic or lethal doses is reassuring evidence that neurotoxicity in humans will be unlikely if the plasma concentrations in animals are at least tenfold greater than those achieved in humans following therapeutic or prophylactic dosing.

Even if drugs have the potential for neurotoxicity in animal models at supra-therapeutic doses, if the human dosing regimen does not achieve pharmacodynamically relevant exposures, and if the safety margins with CNS toxicity in preclinical species are acceptable, then NPAEs should not be expected. For example, although artemisinins are neurotoxic in animal models [17–21], the rate of NPAEs with these agents in patients is very low (1.1%) and despite extensive use of artemisinins globally, NPAEs have not emerged as a significant safety concern [5]. The pre-clinical studies were conducted at supra-therapeutic doses with long durations of exposure and in some cases an oil vehicle was used, which further increased artemisinin drug exposure [22, 23]. There is also evidence that higher exposures are needed to elicit neurotoxicity in primates versus rodents and dogs [22]. In contrast, artemisinins for malaria treatment are given orally, for short durations and at low doses, generally resulting in human drug exposures that would not be expected to cause neurotoxicity [24, 25]. However, concerns still apply to certain populations where intrinsic factors may affect the pharmacokinetics of the drug. For example, oral dispersible artemether-lumefantrine at a dose of 20/120 mg twice daily for 3 days could not be recommended to treat infants weighing < 5 kg with *P. falciparum* malaria, as the artemether and dihydroartemisinin exposures exceeded the preclinical neurotoxicity safety margins [26]. Neurotoxicity may also limit the opportunity to increase artemisinin doses to overcome drug resistance [24]. Note also that following frequent dosing with artesunate in severe malaria, dihydroartemisinin levels in cerebrospinal fluid increased with time while levels in plasma declined, indicating accumulation [27]. Thus, NPAE risk depends on both the neurotoxic potential of a particular agent documented in preclinical studies and the pharmacokinetic/pharmacodynamic profile of the dosing regimen used.

### Clinical adverse events

The MedDRA preferred term classification system is the standard method for assigning adverse events (AEs) to drug treatments used in clinical trials. Two system organ classes are relevant for neuropsychiatric risk: neurological disorders and psychiatric disorders (Table 1).

**Table 1:** MedDRA system organ classes relevant for assessment of drug-related neuropsychiatric risk

Neurological disorders
Peripheral neuropathies, headaches, nervous system neoplasms malignant and unspecified (NEC), spinal cord and nerve root disorders, congenital and peripartum neurological conditions, demyelinating disorders, nervous system neoplasms benign, increased intracranial pressure and hydrocephalus, movement disorders (incl. Parkinsonism), encephalopathies, seizures (incl. subtypes), mental impairment disorders, neurological disorders of the eye, cranial nerve disorders (excl. neoplasms), structural brain disorders, neuromuscular disorders, central nervous system infections and inflammations, sleep disturbances (incl. subtypes), central nervous system vascular disorders
Psychiatric disorders
This is currently based on the Diagnostic and Statistical Manual of Mental Disorders, Fourth Edition, (DSM-IV) and includes changes in physical activity, eating disorders and disturbances, impulse control disorders NEC, cognitive and attention disorders and disturbances, dissociative disorders, somatic symptom and related disorders, sleep disorders and disturbances, psychiatric and behavioural symptoms NEC, disturbances in thinking and perception, schizophrenia and other psychotic disorders, adjustment disorders (incl. subtypes), communication disorders and disturbances, sexual dysfunctions, disturbances and gender identity disorders, developmental disorders NEC, suicidal and self-injurious behaviours NEC, dementia and amnestic conditions, deliria (incl. confusion), manic and bipolar mood disorders and disturbances, anxiety disorders and symptoms, mood disorders and disturbances NEC, personality disorders and disturbances in behaviour, depressed mood disorders and disturbances

*NEC* not elsewhere classifiable

Numerous papers of neuropsychiatric risk following anti-malarial treatment or during prophylaxis have been published, reaching various conclusions and promoting conflicting hypotheses [28]. It is important to note that, although case reports may act as flags for further investigation, on their own they cannot be accepted as evidence of a general drug-related neuropsychiatric effect, particularly for subjective symptoms.

**Fig. 1:**
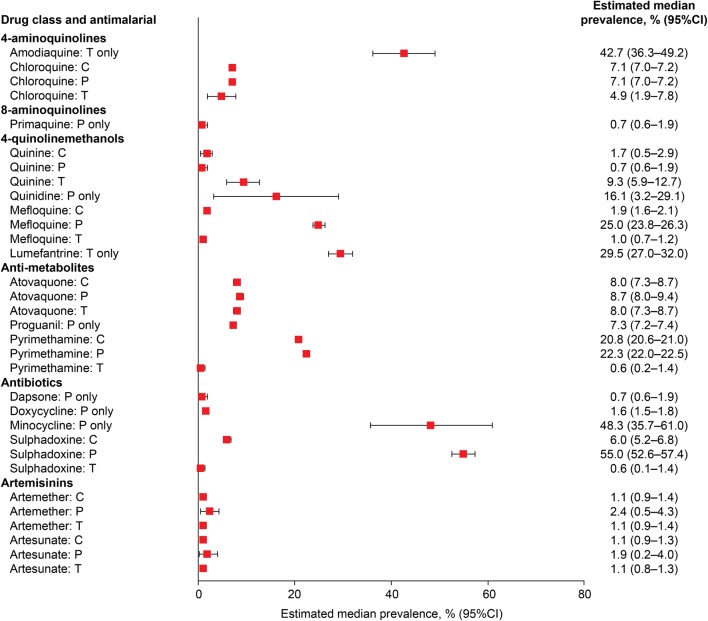
Estimated median prevalence of NPAEs with anti-malarial prophylaxis (P), treatment (T) or combined prophylaxis and treatment (C) [5]

A recent systematic meta-analysis of the published literature on ‘mental and neurological manifestations’ associated with anti-malarial drugs by Bitta et al. [5] provides a rigorous evaluation of the available clinical evidence. Specific psychiatric and neurological symptoms were examined for six anti-malarial drug classes across studies of treatment and prophylaxis (Fig. 1).

It can be seen immediately that there is no relationship between the prevalence of pooled NPAEs and the class of anti-malarial, except perhaps for the artemisinins, where prevalence is low for both artemether and artesunate. The lowest pooled prevalence of NPAEs was with the 8-aminoquinoline primaquine and the antibiotic dapsone and the highest was noted for the antibiotic minocycline and the 4-aminoquinoline amodiaquine. It can also be seen that for some drugs (mefloquine, pyrimethamine, sulphadoxine), the prevalence of events is far higher in prophylaxis studies than during malaria treatment, whereas for other drugs (chloroquine, atovaquone, artemether, artesunate) there is little difference between prophylaxis and treatment, and for quinine, prevalence is higher during treatment than for prophylaxis. Thus, we can conclude that there is no clear pattern of predicting NPAEs based on class or clinical indication, but that the risk depends on the specific neurotoxic potential and pharmacokinetic/pharmacodynamic properties of the drug (i.e. whether toxicity is related to maximum drug concentrations or to cumulative exposure) under the different dosing regimens for treatment and prophylaxis.

The meta-analysis described above allows us to compare different drugs and identify outliers in treatment studies, which either by their nature or frequency, are suggestive of drug-related NPAEs. However, this introduces limitations in terms of making comparisons across different patient populations that may have different risk profiles for neuropsychiatric symptoms, be infected with different or multiple *Plasmodium* species, possess different immunity profiles, or have variations in transmission settings and socio-economic conditions that might affect non-drug related NPAE risk.

## Confounding factors in evaluating NPAE risk

### Malaria effects versus drug effects in patients

Malaria itself can produce varied and sometimes severe neuropsychiatric symptoms, the most serious being associated with cerebral malaria, including abnormal behaviour, impairment of consciousness, seizures, coma, or other neurologic abnormalities [29]. However, even in milder disease, symptoms commonly include high fever, headache, fatigue and confusion [29, 30]. Previous malaria episodes can also lead to long-term cognitive, behavioural and psychological symptoms [31–39]. Thus, evaluating the relative contributions of drug and disease to NPAEs following anti-malarial treatment is very difficult, and the same data can be interpreted differently, leading to a range of opinions.

In treatment studies, which are typically conducted across a single study population, the relative NPAE risk of a new anti-malarial versus the comparator is evaluated; the comparator is usually determined by the relevant National Malaria Treatment Guidelines. However, as most new anti-malarial drugs are administered in combination, the contribution of each component of the regimen to the incidence of adverse events cannot be determined. In the case of *P. vivax* relapse prevention, new drug regimens are compared to placebo/chloroquine or placebo/artemisinin-based combination therapy (ACT) or the standard-of-care primaquine/chloroquine or primaquine/ACT. Thus, any adverse event signals outside the frequencies observed with these comparators would be concerning.

### Prophylaxis versus treatment

Prophylaxis studies remove the confounding effect of malaria symptomatology and allow a placebo-controlled comparison with a reference group. However, the dosing regimens and drug pharmacokinetics in prophylaxis are very different to those for treatment. In treatment, higher doses are generally given over a shorter time period, usually no more than 3 days, with the possible risk of adverse events related to maximum concentrations achieved. In contrast, prophylaxis generally involves lower doses regularly administered over a protracted time period, often months, with the risk of adverse events secondary to drug accumulation. For example, in the Bitta et al. meta-analysis outlined above, the difference between the NPAE risk for mefloquine in prophylaxis (25%) versus treatment (1%) was stark (Fig. 1). The increased frequency of NPAEs with mefloquine in prophylaxis is likely derived from the specific pharmacokinetics of the drug during the prophylactic dosing regimen, i.e. its ability to cross the blood–brain barrier, distribute and accumulate into cerebral tissue [40, 41], combined with its underlying potential for neurotoxicity at high doses, as suggested in pre-clinical studies [4, 42–44]. Drugs that do not accumulate following multiple dosing in prophylaxis may actually have a lower frequency of adverse events than during treatment, for example, quinine (Fig. 1). Thus, data from prophylaxis studies cannot be extrapolated to predict NPAE risk during malaria treatment or vice versa.

### Underlying neuropsychiatric risk

The population that receives malaria treatment or prophylaxis may not have the same risk for neuropsychiatric symptoms as the general population. For example, as mentioned above, previous malaria can lead to ongoing psychiatric and behavioural issues [31–39]. Exposure to insecticides used for malaria prevention, including dichlorodiphenyltrichloroethane (DDT), and pesticides for crop protection in rural areas, have also been suggested as a source of neurotoxicity [45–47]. Poverty and malnutrition [48], as well as population displacement and conflict can also predispose to neuropsychiatric events [49], and are also risk factors for malaria [50].

In the case of prophylaxis, travel poses a number of potential neurological risks. Changes to routine and situation can trigger neuropsychiatric symptoms, for example by disruption of normal circadian rhythms or separation from support networks [51], as can certain foods and infections that travellers may be exposed to [52, 53].

The most controversial example of data generated on malaria prophylaxis is from studies in military personnel. Malaria is a serious risk to the operational effectiveness of a military force. Thus, there is a compelling motivation to identify the most suitable prophylactic agents for use in military deployments to malaria endemic regions. Moreover, malaria is a potentially fatal disease in non-immune individuals and long-term sequelae may afflict survivors. Even where studies have compared a non-immune military population deployed to a malaria endemic region against a ‘control’ of either an untreated resident population, or a non-deployed military population [54], it is important to understand the different underlying risk for neuropsychiatric findings in the compared populations.

To evaluate NPAE risk in soldiers given anti-malarial prophylaxis (deployed or non-deployed, with or without combat), we must first understand the underlying neuropsychiatric risk in military personnel versus civilians or in deployed versus non-deployed military personnel. If military personnel have a greater underlying risk for neuropsychiatric events than the general population, then trying to determine which NPAEs are drug-related, potentially over several months of treatment, becomes problematic, particularly if the underlying risk increases with deployment, i.e. over the same period that anti-malarial prophylaxis is taken.

In general, examination of large military health databases indicates that serving military personnel are at greater risk of neuropsychiatric symptoms, particularly depression, post-traumatic stress disorder and other internalizing conditions than the civilian population [55–67]. This may result from a higher frequency of pre-enlistment mental health issues in military personnel, particularly soldiers, versus those who choose alternative occupations [56, 61, 62]. Deployment itself, particularly if associated with combat, affects neuropsychiatric risk [58, 61, 63–66, 68–71]. Thus, it might be expected that NPAEs occur more frequently in military personnel than the general population because of a higher underlying incidence of neuropsychiatric disorders. However, it is also possible that a drug which is associated with rare NPAEs in the general population may elicit such events more frequently in a more vulnerable population with a greater prevalence of pre-existing mental health issues, and/or a population subjected to severe stress, such as war-like military deployment [2, 72]. It seems reasonable, therefore, that NPAE risk should be evaluated prospectively and separately in specific populations (travellers, malaria endemic residents, deployed military, non-deployed military), as the results cannot necessarily be extrapolated between these groups.

## Tafenoquine pre-clinical neurotoxicity assessment

The activity–structure relationships for the 8-aminoquinolines have been studied, with methyl substitution at position 4 of the quinoline ring conferring protection against neurotoxicity [73]. As tafenoquine is 4-methyl-substituted, neurotoxicity would not be anticipated a priori.

Several pre-clinical studies to assess the potential CNS effects of tafenoquine have been conducted, including histopathological assessments in single- and repeat-dose studies in mice, rats and dogs, and detailed assessments of both neurobehavioural function and neurohistopathology in both single- and repeat-dose studies in rats [74]. Across these studies, there was no evidence of neurotoxicity with tafenoquine [74]. Additionally, distribution of radiolabelled tafenoquine into rat brain following a single oral dose of up to 25 mg/kg showed poor penetration with brain concentrations of < 1% of the dose and low concentrations relative to other body tissues/organs [74].

A comprehensive battery of preclinical neurotoxicological tests has been conducted on tafenoquine at supra-therapeutic and lethal doses in rats [75]. Briefly, the maximum tolerated single dose of tafenoquine in rats was identified as 500 mg/kg, with non-neurological toxicities (gastrointestinal and haematological) found to be dose limiting. Based on these findings, neurobehavioural, histopathologic and toxicokinetic studies were conducted using single doses of 125, 250 or 500 mg/kg tafenoquine or placebo [75]. The standard functional observation battery was performed pre-test, 0.5, 3, 6, 24 and 48 h post-dose. Motor activity was assessed using the number of beam breaks observed, and at doses > 9-fold higher than the clinical exposure, motor activity was reduced at 48 h following tafenoquine dosing [75]. Across all other assessments, there were no significant findings with tafenoquine relative to control rats [75]. Histopathological investigations were conducted in all animals dosed with 500 mg/kg. There was no evidence of any neuropathological changes in brain sections and no evidence of neurodegeneration or other morphological abnormalities [75]. There were no abnormalities identified in the gracile nucleus, previously cited as a potential target for drug toxicity [42, 75]. In conclusion, using experiments in rats that conclusively demonstrated neurotoxicity with mefloquine [42], there was no evidence of neurotoxicity with tafenoquine [75].

Although no pre-clinical neurotoxicity experiments were conducted in primates, several pharmacokinetic/pharmacodynamic studies of tafenoquine were conducted in Rhesus macaques. These studies were overseen by certified veterinarians and there was no evidence of any neurobehavioural disturbance or clinical evidence of neurotoxicity [76–79].

## Tafenoquine for *P. vivax* relapse prevention

### High-dose tafenoquine monotherapy versus chloroquine/primaquine

**Fig. 2:**
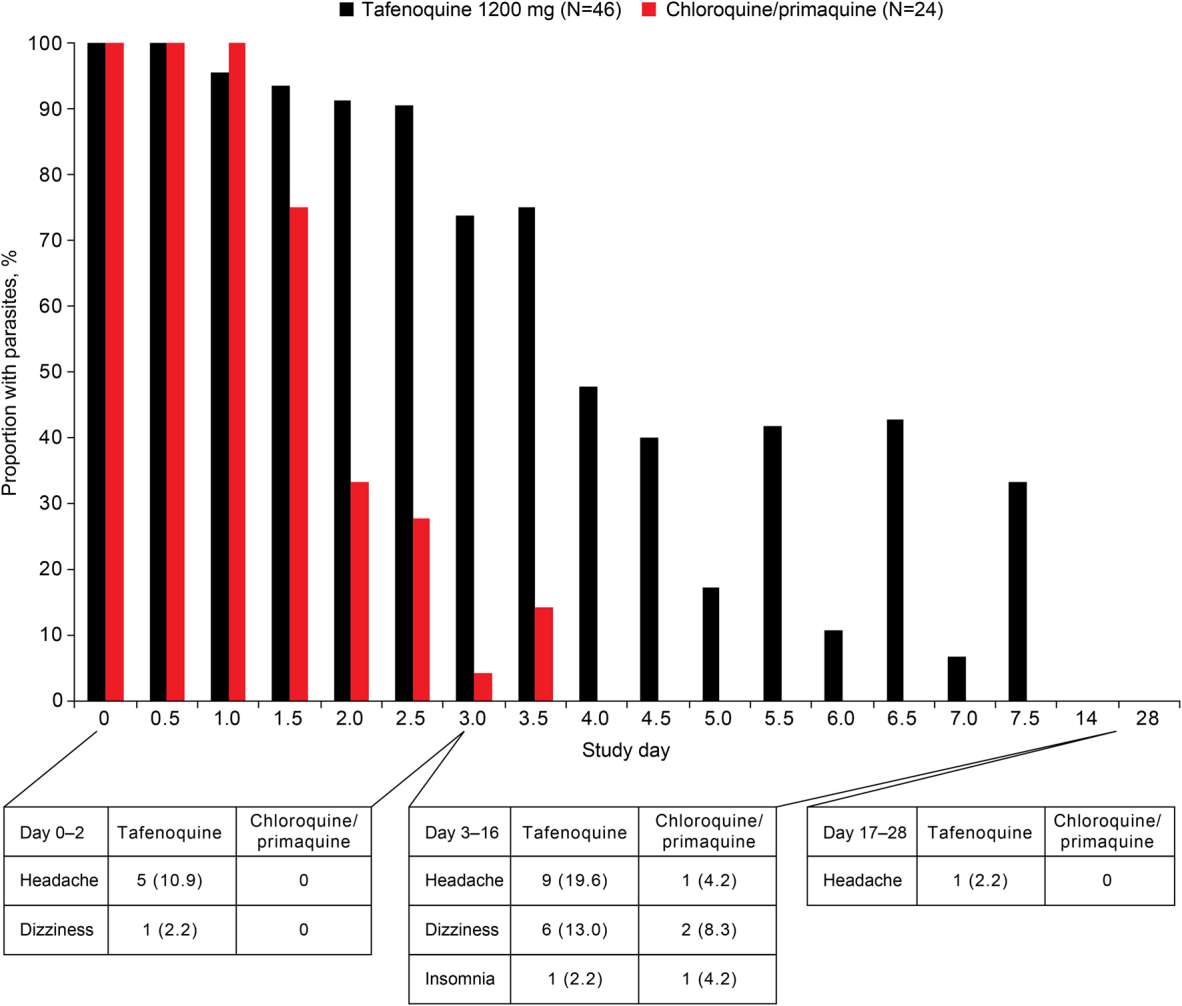
Neuropsychiatric adverse events occurring with high-dose tafenoquine monotherapy or chloroquine/primaquine versus *P. vivax* parasitaemia [80]

Tafenoquine was previously investigated as monotherapy in *P. vivax* relapse prevention [80]. In a randomized, active-control, double-blind trial (Bangkok, Thailand), *P. vivax* patients were randomized to tafenoquine 400 mg once daily for 3 days (N = 46) or standard 1500 mg (base) total dose chloroquine given over 3 days plus primaquine 15 mg daily for 14 days (N = 24) [80]. This high monotherapy dose was abandoned as it had slow clearance of malaria parasites coupled with an unacceptable rate of drug-induced methaemoglobinemia. Hence, the further clinical development of tafenoquine was conducted in combination with a blood schizonticide. In terms of NPAEs, headache and dizziness were more common in the tafenoquine monotherapy group versus chloroquine/primaquine (Fig. 2). Although this could conceivably be a drug-related nervous system adverse effect, the headache and dizziness were more likely caused by slow parasite clearance under tafenoquine monotherapy: malaria symptoms persisted for longer in this group versus the chloroquine/primaquine group (Fig. 2) [80].

### High-dose tafenoquine/chloroquine

Initial studies of tafenoquine/chloroquine in *P. vivax* relapse prevention examined tafenoquine doses of 300 mg/day for 7 days, 600 mg/day for 3 days and 600 mg single dose compared to chloroquine plus primaquine 15 mg/day for 14 days (Table 2) [81]. Even at the highest tafenoquine dose (2100 mg cumulative), NPAEs occurred less frequently overall than with primaquine/chloroquine and were confined to vertigo and headache (Table 2). There was a trend for both vertigo and headache to occur more frequently at the higher tafenoquine doses. However, at the tafenoquine 600 mg single-dose, the incidence of vertigo was similar to that of primaquine/chloroquine and the incidence of headache was lower at all tafenoquine doses versus primaquine/chloroquine (Table 2).

**Table 2:** Frequency of NPAEs with high-dose tafenoquine/chloroquine in vivax malaria patients [81]

Adverse event, n (%)	Tafenoquine 300 mg q.d. × 7 days (N = 18)	Tafenoquine 600 mg q.d. × 3 days (N = 19)	Tafenoquine 600 mg single dose (N = 18)	Primaquine 15 mg q.d. × 14 days (N = 12)
Any NPAE	8 (44)	8 (42)	4 (22)	6 (50)
Vertigo	8 (44)	8 (42)	4 (22)	3 (25)
Headache	4 (22)	4 (21)	2 (11)	4 (33)

### Single-dose tafenoquine/chloroquine

Based on the need to co-administer a blood schizonticide, three comparative clinical studies have been conducted with single-dose tafenoquine plus standard 3-day chloroquine in *P. vivax* relapse prevention in centres across Asia, South America and Africa.

- DETECTIVE Phase 2b (TAF112582 part 1): a multicentre, phase 2b, double-blind, placebo-controlled, randomized, dose-selection study of single-dose tafenoquine (50, 100, 300, 600 mg) plus standard 3-day chloroquine versus placebo plus standard 3-day chloroquine or primaquine 15 mg for 14 days plus standard 3-day chloroquine [9].
- DETECTIVE Phase 3 (TAF112582 part 2): a multicentre, phase 3 pivotal, double-blind, double-dummy, randomized placebo-controlled clinical trial of single-dose tafenoquine 300 mg plus standard 3-day chloroquine versus placebo plus standard 3-day chloroquine or primaquine 15 mg for 14 days plus standard 3-day chloroquine [10].
- GATHER (TAF116564): a multicentre, phase 3 supportive, double-blind, double-dummy, parallel group, randomized trial of single-dose tafenoquine 300 mg plus standard 3-day chloroquine versus primaquine 15 mg for 14 days plus standard 3-day chloroquine [11].

There are some key aspects that need to be highlighted in the assessment of NPAE risk with tafenoquine in *P. vivax* relapse prevention in these studies. Firstly, patients were infected with *P. vivax* malaria and symptomatic. Secondly, tafenoquine was co-administered with standard 3-day chloroquine, and so evaluation of tafenoquine NPAEs is hampered by the absence of a true placebo arm with comparisons having to be made against either chloroquine alone or primaquine/chloroquine. Lastly, in all three clinical trials, *P. vivax* recurrence after day 29 resulted in retreatment with primaquine/chloroquine and so adverse events recorded after this point include adverse events associated with recurrence of malaria as well as those associated with the rescue therapy. As relapse was more frequent in the chloroquine alone group than with either of the 8-aminoquinolines, adverse events after day 29 were consequently more frequent with placebo/chloroquine than for the 8-aminoquinoline arms. Thus, only adverse events occurring before day 29 should be considered for comparison to the placebo/chloroquine arm.

To further investigate whether an NPAE signal might be present in the safety database from these studies, an expanded definition of NPAEs was applied. As well as including the standard terms included under ‘nervous system disorders’ and ‘psychiatric disorders’, the expanded definition added labyrinthitis, vertigo, vestibular disorder, asthenia, fatigue, and alcohol intolerance to include terms with possible reference to vestibulocochlear function, which has its seat in the brainstem. This was considered important, considering the eighth cranial nerve appeared to have been affected adversely by some earlier quinoline molecules [82, 83].

#### Tafenoquine/chloroquine dose ranging

The DETECTIVE 2b study was designed to select the clinical regimen for tafenoquine/chloroquine in *P. vivax* relapse prevention and so was conducted in malaria patients [9]. The most common NPAEs with tafenoquine/chloroquine at all doses were headache and dizziness, though at the highest tafenoquine dose of 600 mg, these occurred less frequently than with placebo/chloroquine. All other NPAEs were infrequent (Table 3). There was no evidence of any dose–response for any NPAE (Table 3).

**Table 3:** Frequency of NPAEs in *P. vivax* malaria patients given tafenoquine/chloroquine, primaquine/chloroquine or placebo/chloroquine [9]

Adverse event, n (%)	TQ + CQ				PQ + CQ (N = 50)	Placebo + CQ (N = 54)
	50 mg (N = 55)	100 mg (N = 57)	300 mg (N = 57)	600 mg (N = 56)		
Any nervous system disorder	17 (31)	17 (30)	13 (23)	16 (29)	17 (34)	21 (39)
Headache	14 (25)	17 (30)	10 (18)	16 (29)	14 (28)	20 (37)
Dizziness	7 (13)	2 (4)	5 (9)	4 (7)	5 (10)	5 (9)
Migraine	0	0	0	0	0	1 (2)
Tremor	0	0	1 (2)	1 (2)	0	0
Paraesthesia	0	0	0	1 (2)	0	0
Sciatica	0	1 (2)	0	0	0	0
Burning sensation	1 (2)	0	0	0	0	0
Syncope	0	0	0	0	1 (2)	0
Dysaesthesia	0	0	0	0	1 (2)	0
Any psychiatric disorder	2 (4)	3 (5)	5 (9)	3 (5)	1 (2)	1 (2)
Insomnia	2 (4)	3 (5)	5 (9)	3 (5)	3 (6)	1 (2)
Tic	0	0	0	1 (2)	0	0
Expanded terms^a^						
Asthenia	5 (9)	4 (7)	1 (2)	5 (9)	0	0
Fatigue	0	1 (2)	1 (2)	0	0	0

*CQ* chloroquine, *PQ* primaquine, *TQ* tafenoquine

^a^Expanded terms: labyrinthitis, vertigo, vestibular disorder, asthenia, fatigue, and alcohol intolerance were also considered with possible reference to vestibulocochlear function

#### Tafenoquine/chloroquine versus placebo/chloroquine

**Fig. 3:**
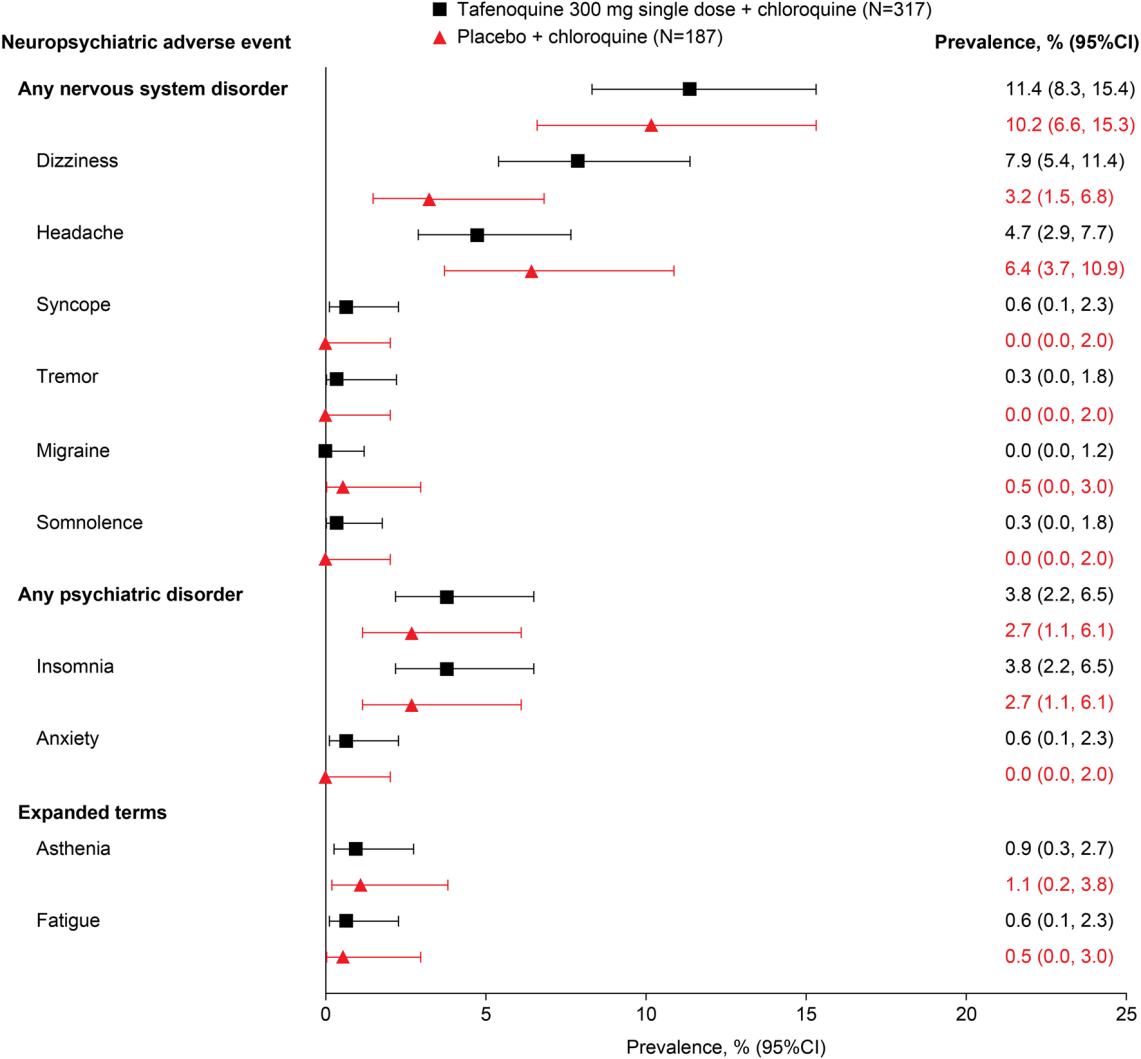
Neuropsychiatric adverse events occurring before day 29 in *P. vivax* patients given tafenoquine/chloroquine versus placebo/chloroquine [9, 10]

DETECTIVE Phase 2b and DETECTIVE Phase 3 were placebo-controlled randomized studies designed as a seamless Phase 2b/3 programme, with similar protocols allowing data to be pooled for the single tafenoquine 300 mg therapeutic dose [9, 10]. All patients in the tafenoquine and placebo groups received 3-day chloroquine started on day 1; tafenoquine or placebo was given on day 1 or 2. Across the two studies, the overall prevalence of any nervous system disorders occurring before day 29 of the study was 11.4% (36/317) with tafenoquine/chloroquine and 10.2% (19/187) with placebo/chloroquine; psychiatric adverse events occurred in 3.8% (12/317) and 2.7% (5/187) of patients, respectively (Fig. 3).

For individual adverse events, only headache and dizziness occurred in more than 5% of patients, with a trend for a higher incidence of dizziness and lower incidence of headache with tafenoquine/chloroquine versus placebo/chloroquine, though 95% CIs overlapped in both cases (Fig. 3). Psychiatric adverse events were confined to insomnia and anxiety at low prevalence in both treatment arms (Fig. 3). In these well-controlled randomized studies, none of the NPAEs identified led to study withdrawal or therapy interruption and there were no NPAEs classified as serious or severe. These results suggest a favourable NPAE profile for tafenoquine/chloroquine, similar to that of chloroquine alone, with a low prevalence of events of mild-to-moderate severity.

#### Tafenoquine/chloroquine versus primaquine/chloroquine

**Fig. 4:**
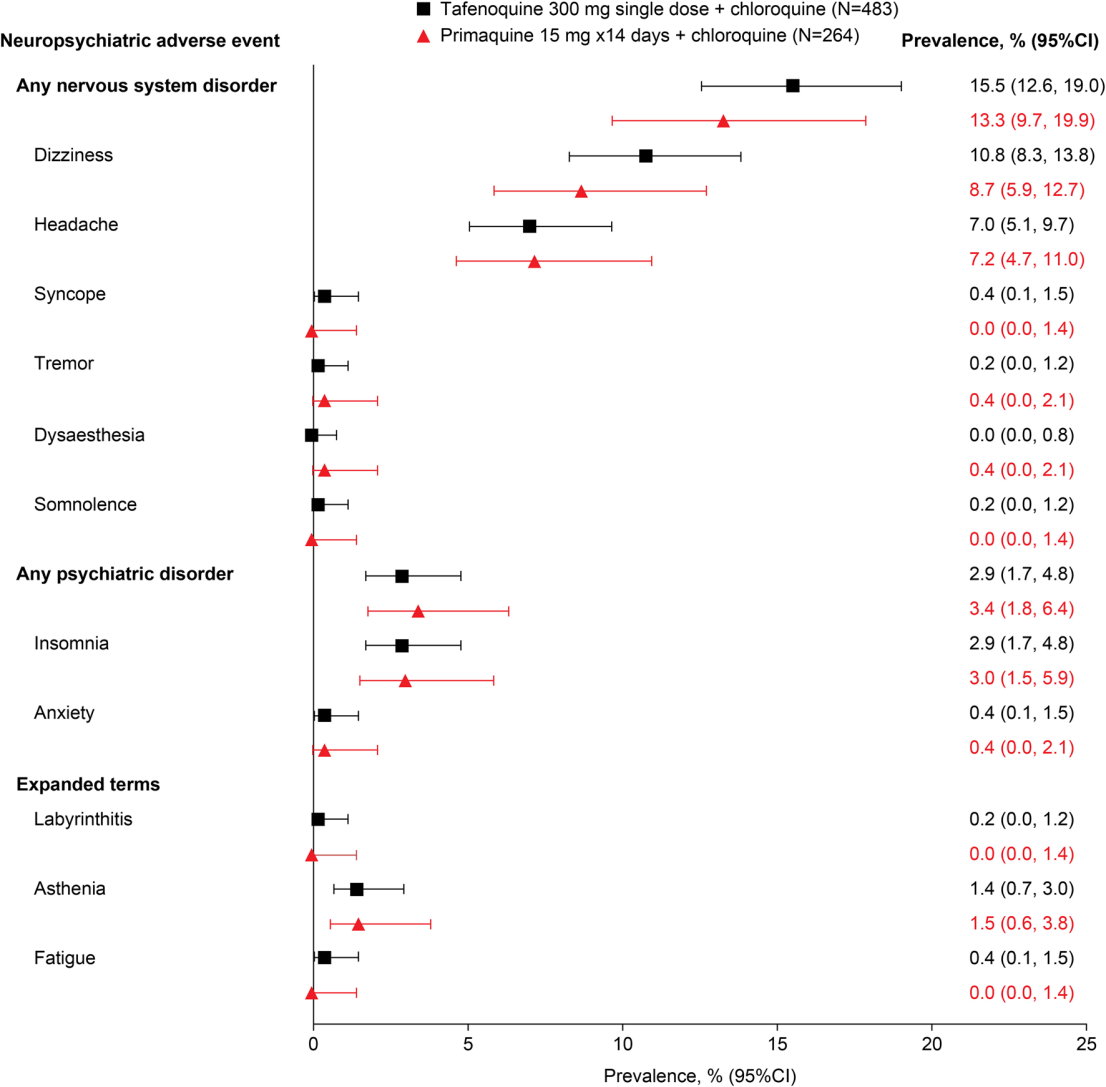
Neuropsychiatric adverse events occurring before day 29 in *P. vivax* patients given tafenoquine/chloroquine versus primaquine/chloroquine [9–11]

Primaquine/chloroquine was included as a comparator in the DETECTIVE Phase 2b, DETECTIVE Phase 3 and GATHER studies [9–11]. All three studies had similar protocols and all used the standard primaquine dose of 15 mg/day for 14 days. Across the three studies, the overall prevalence of any nervous system disorders occurring before day 29 was 15.5% (75/483) with tafenoquine/chloroquine and 13.3% (35/264) with primaquine/chloroquine; psychiatric adverse events occurred in 2.9% (14/483) and 3.4% (9/264) of patients, respectively (Fig. 4).

As was seen for the comparison versus placebo, for both tafenoquine/chloroquine and primaquine/chloroquine, only dizziness and headache occurred in > 5% of patients, and with a similar prevalence for both comparators (Fig. 4). Psychiatric adverse events were confined to insomnia and anxiety at low prevalence in both treatment arms (Fig. 4). None of the NPAEs identified led to study withdrawal or therapy interruption and there were no NPAEs classified as serious or severe.

When examining the frequency of all adverse events occurring over the 6-month study duration, there was no evidence of any increased risk for NPAEs with tafenoquine/chloroquine versus primaquine/chloroquine (Table 4). As would be expected given the longer follow up period, the frequency of NPAEs in both arms was higher after 6 months of follow up (Table 4) versus day 29 (Fig. 4), but there were no differences between the treatment groups. Nervous system adverse events occurred in 21.7% (105/483) of patients in the tafenoquine/chloroquine group and 22.7% (60/264) in the primaquine/chloroquine group; psychiatric adverse events occurred in 3.1% (15/483) and 4.5% (12/264), respectively. None of the NPAEs identified in the tafenoquine/chloroquine group led to study withdrawal or therapy interruption and there were no NPAEs classified as serious or severe. These results suggest an NPAE profile for tafenoquine/chloroquine similar to that of primaquine/chloroquine, with a low prevalence of NPAEs of mild-to-moderate severity.

**Table 4:** Frequency of all NPAEs occurring with tafenoquine/chloroquine versus primaquine/chloroquine (day 1 to day 180) [9–11]

Event—n (%) patients	Grade^a^	Integrated safety summary	
		Tafenoquine (N = 483)	Primaquine (N = 264)
Nervous system disorders	Any	105 (22)	60 (23)
	Grade 1	78 (16)	38 (14)
	Grade 2	26 (5)	19 (7)
	*Grade 3*	*0*	*2 (< 1)*
	NA	1 (< 1)	1 (< 1)
Headache	Any	64 (13)	40 (15)
	Grade 1	45 (9)	22 (8)
	Grade 2	18 (4)	16 (6)
	*Grade 3*	*0*	*2 (< 1)*
	NA	1 (< 1)	0
Dizziness	Any	59 (12)	30 (11)
	Grade 1	52 (11)	25 (9)
	Grade 2	7 (1)	4 (2)
	NA	0	1 (< 1)
Migraine	Any	3 (< 1)	1 (< 1)
	Grade 1	2 (< 1)	0
	Grade 2	1 (< 1)	1 (< 1)
Syncope	Any	2 (< 1)	1 (< 1)
	Grade 1	1 (< 1)	1 (< 1)
	Grade 2	1 (< 1)	0
Balance disorder	Any	1 (< 1)	0
	Grade 2	1 (< 1)	0
Somnolence	Any	1 (< 1)	0
	Grade 2	1 (< 1)	0
Tremor	Any	1 (< 1)	1 (< 1)
	Grade 1	1 (< 1)	1 (< 1)
Burning sensation	Any	0	1 (< 1)
	Grade 1	0	1 (< 1)
Dysaesthesia	Any	0	1 (< 1)
	Grade 1	0	1 (< 1)
Hypoaesthesia	Any	0	1 (< 1)
	Grade 2	0	1 (< 1)
Psychiatric disorders	Any	15 (3)	12 (5)
	Grade 1	9 (2)	7 (3)
	Grade 2	6 (1)	5 (2)
Insomnia	Any	15 (3)	8 (3)
	Grade 1	9 (2)	4 (2)
	Grade 2	6 (1)	4 (2)
Anxiety	Any	2 (< 1)	3 (1)
	Grade 1	0	2 (< 1)
	Grade 2	2 (< 1)	1 (< 1)
Depression	Any	0	1 (< 1)
	Grade 1	0	1 (< 1)
Expanded definition of NPAEs			
Asthenia	Any	8 (2)	5 (2)
	Grade 1	7 (1)	2 (< 1)
	Grade 2	1 (< 1)	3 (1)
Fatigue	Any	3 (< 1)	0
	Grade 1	3 (< 1)	0
Labyrinthitis	Any	2 (< 1)	0
	Grade 1	2 (< 1)	0
Alcohol intolerance	Any	1 (< 1)	0
	Grade 1	1 (< 1)	0
Vertigo	Any	3 (< 1)	1 (< 1)
	Grade 1	2 (< 1)	1 (< 1)
	Grade 2	1 (< 1)	0
Vestibular disorder	Any	1 (< 1)	0
	Grade 1	1 (< 1)	0

Data are from the DETECTIVE phase 2b, DETECTIVE phase 3, and GATHER trials (safety population). Vivax malaria patients were treated with chloroquine plus either single-dose tafenoquine 300 mg or primaquine 15 mg for 14 days. Adverse events of Grade 3 and above in any treatment group are italicized

Note that this includes adverse events that were associated with recurrences after day 29. Subjects that experienced recurrence received further treatment with primaquine. Thus, the comparison of adverse events before day 29 provides a more reliable indicator of potential differences caused by drug treatment (see Fig. 4)

^a^Common Terminology Criteria for Adverse Events v4.0

## Tafenoquine safety database analysis

An analysis of all tafenoquine clinical safety data available to GlaxoSmithKline (all studies safety database) was conducted to support review by the US Food and Drug Administration [74]. This included healthy volunteers, *P. vivax* patients receiving tafenoquine/chloroquine and those receiving tafenoquine prophylaxis. Overall, 4131 tafenoquine-exposed subjects were included compared to 792 who received placebo/chloroquine or placebo [74]. Comparing across these results is difficult as treatment and prophylaxis studies would be expected to have differing adverse event profiles, both in the active and the placebo arms. However, overall there was no evidence that tafenoquine/chloroquine 300 mg for ≤ 3 days had an increased risk of NPAEs versus placebo (Table 5). There were no serious, severe or medically important NPAEs (0/807) identified in any of the studies in which tafenoquine was given at 300 mg for ≤ 3 days.

**Table 5:** Adverse events classified as nervous system disorders or psychiatric disorders reported across the entire tafenoquine development programme classified by total tafenoquine dose [74]

Adverse event, n (%)	All placebo^a^ (N = 794)	Total TQ 300 mg ≤ 3 days (N = 807)	Total TQ > 300 mg ≤ 3 days (N = 1482)	Total TQ > 300 mg > 3 days (N = 1445)	All TQ^b^ (N = 4129)
Any nervous system disorder	170 (21)	142 (18)	240 (16)	269 (19)	734 (18)
Headache	149 (19)	98 (12)	164 (11)	211 (15)	544 (13)
Migraine	4 (< 1)	3 (< 1)	0	3 (< 1)	8 (< 1)
Sinus headache	1 (< 1)	0	0	4 (< 1)	4 (< 1)
Tension headache	0	0	0	2 (< 1)	2 (< 1)
Head discomfort	0	0	1 (< 1)	0	1 (< 1)
Visual field defect	0	0	0	1 (< 1)	1 (< 1)
Lethargy	0	0	28 (2)	28 (2)	56 (1)
Somnolence	1 (< 1)	3 (< 1)	21 (1)	1 (< 1)	25 (< 1)
Amnesia	0	0	0	1 (< 1)	1 (< 1)
Depressed level of consciousness	0	1 (< 1)	0	0	1 (< 1)
Disturbance in attention	0	0	1 (< 1)	0	1 (< 1)
Dysgeusia	1 (< 1)	0	17 (1)	1 (< 1)	18 (< 1)
Paraesthesia	0	0	3 (< 1)	4 (< 1)	8 (< 1)
Hypoaesthesia	1 (< 1)	0	3 (< 1)	1 (< 1)	4 (< 1)
Hyperaesthesia	0	0	0	1 (< 1)	2 (< 1)
Burning sensation	0	0	0	0	1 (< 1)
Coordination abnormal	0	0	0	2 (< 1)	2 (< 1)
Balance disorder	0	1 (< 1)	0	0	1 (< 1)
Dizziness	24 (3)	62 (8)	56 (4)	33 (2)	171 (4)
Syncope	0	2 (< 1)	1 (< 1)	2 (< 1)	5 (< 1)
Presyncope	0	0	0	1 (< 1)	1 (< 1)
Dizziness postural	1 (< 1)	0	0	0	0
Loss of consciousness	1 (< 1)	0	0	0	0
Tremor	0	1 (< 1)	1 (< 1)	2 (< 1)	4 (< 1)
Muscle contractions involuntary	0	0	1 (< 1)	0	1 (< 1)
Sciatica	0	0	0	2 (< 1)	3 (< 1)
Post herpetic neuralgia	0	0	1 (< 1)	1 (< 1)	2 (< 1)
Trigeminal neuralgia	0	0	0	1 (< 1)	1 (< 1)
Any psychiatric disorder	8 (1)	16 (2)	19 (1)	37 (3)	79 (2)
Insomnia	8 (1)	15 (2)	12 (< 1)	15 (1)	48 (1)
Abnormal dreams	0	1 (< 1)	0	6 (< 1)	7 (< 1)
Sleep disorder	0	0	0	3 (< 1)	3 (< 1)
Nightmare	0	0	0	2 (< 1)	2 (< 1)
Agitation	0	0	0	2 (< 1)	2 (< 1)
Anxiety	0	2 (< 1)	0	0	2 (< 1)
Anxiety disorder	0	0	0	2 (< 1)	2 (< 1)
Irritability	0	0	1 (< 1)	0	2 (< 1)
Neurosis	0	0	0	1 (< 1)	1 (< 1)
Panic attack	0	0	0	1 (< 1)	1 (< 1)
Psychotic disorder	0	0	1 (< 1)	0	1 (< 1)
Stress	0	0	0	1 (< 1)	1 (< 1)
Euphoric mood	0	0	1 (< 1)	2 (< 1)	3 (< 1)
Depressed mood	0	0	1 (< 1)	1 (< 1)	2 (< 1)
Depression	0	0	0	2 (< 1)	2 (< 1)
Bipolar disorder	0	0	0	1 (< 1)	1 (< 1)
Disinhibition	0	0	1 (< 1)	0	1 (< 1)
Mood altered	0	0	1 (< 1)	0	1 (< 1)
Alcoholic hangover	0	0	0	1 (< 1)	1 (< 1)
Tic	0	0	1 (< 1)	0	1 (< 1)

^a^The placebo group includes healthy volunteers treated with placebo and *P. vivax* subjects treated with chloroquine alone in *P. vivax* relapse prevention

^b^Also includes 392 subjects who received < 300 mg total dose and 3 subjects who received 300 mg total dose > 3 days

The tafenoquine all studies safety database (Table 5), plus data from five previous clinical pharmacology studies conducted by the US Army were reviewed for serious or severe psychiatric disorders or medically important events. Nine subjects were identified, five with depression, three with psychosis and one with suicidal behaviour (Table 6). Only one case of depression occurred in a *P. vivax* patient; this individual had a history of depression. Three of the other four subjects with depression had no previous history of psychiatric illness or predisposing medical conditions. However, all four subjects with psychosis or suicidal behaviour had relevant previous psychiatric histories. Whether these events represented the underlying psychiatric conditions of the subjects or whether their symptoms were triggered or worsened by tafenoquine cannot be conclusively determined. Thus, for tafenoquine prophylaxis the precautionary recommendation is that the drug is not given to those with a history of psychotic disorders, or who currently have psychotic symptoms, including hallucinations (seeing or hearing things that are not really there), delusions (false or strange thoughts or beliefs), or disorganized thinking or behaviour [84]. This is not the case for single-dose tafenoquine for *P. vivax* relapse prevention, although it is recommended that a careful medical history be taken to identify any pre-existing mental health problems [85].

**Table 6:** Details of subjects with severe, serious or medically important psychiatric adverse events following tafenoquine [74]

Study identification (indication) [refs.]	Study design	Dose	Event preferred term (severity or verbatim terms)	Onset	Duration	Resolution	Relationship to study drug by the Investigator	Intervention/action taken	Medical history
SB252263/043 (prophylaxis) [89]	R, DB, PC	750 mg cumulative	Suicidal behaviour (associated with alcohol intoxication)^a^	Day 8	1 day after tafenoquine discontinued	Resolved	Related (chloroquine co-suspect)^b^	Hospitalized and required corrective therapy (intervention unknown)/withdrawn due to unstable mental state	Family reported history of marital difficulties and previous suicide threat; no other relevant history or concomitant medications reported at screening
SB252263/050 subject A (volunteers) [90]	R, DB, PC	350 mg single dose	Acute psychotic episode (severe)	Day 24	25 days	Recovered	Possibly related	Hospitalized following progressive emotional distress	Two previous episodes of psychosis (not disclosed at screening)
SB252263/050 subject B (volunteers) [90]	R, DB, PC	500 mg single dose	Psychotic episode (severe)	Day 8	9 days	Recovered	Remotely related	Hospitalized for a pre-scheduled psychiatric admission	Recent diagnosis of schizophrenia (not disclosed at screening)
SB252263/014 (volunteers) [unpublished]	R, O, PG	1200 mg cumulative	Paranoid hallucinotic psychosis (serious)	Day 27	3 days	Not resolved	Unrelated	Hospitalized and treated with olanzapine and lorazepam/none	History of “hallucinotic psychosis” 6 months earlier (not disclosed at screening); no obvious signs of psychosis at screening; negative drug screen
TAF112582 part 1 (*P. vivax* relapse prevention) [9]	R, DB, PC	600 mg single dose	Depressed mood	Day 6	Unknown	Resolved	Unrelated (chloroquine co-suspect)^b^	Hospitalized (on day 88 for 2 days) with nausea, epigastric pain, diarrhoea, and depression; treated with fluoxetine; consulted with psychiatric specialist (findings unknown)	History of depression but no suicidal tendencies; irregular psychiatric consults; frequent but irregular use of diazepam (10 mg)
TAF114582 (volunteers) [91]	R, SB, PC, AC	600 mg single dose	Depressed mood (mild)	Day 4	3 days	Resolved	Related	None	No relevant past medical history or concomitant medications were reported; at the time of the event, subject also reported abdominal pain, diarrhoea, and palpitations
SB252263/033 (prophylaxis) [7]	R, DB, AC	1200 mg cumulative	Depression (moderate)	Day 24	87 days	Resolved	Related	Required corrective therapy (paroxetin)/withdrawn from study	Closed head injury 3 years prior to study
SB252263/057 subject B (volunteers) [92]	R, DB, PC	1600 mg cumulative	Depressed mood (mild)	Day 37	15 days	Resolved	Unlikely related	None	No relevant past history; treated for a UTI with sulfamethoxazole starting on day 13 (co-suspect)
SB252263/057 subject A (volunteers) [92]	R, DB, PC	5200 mg^c^ cumulative	Bipolar depression (mild); depression (mild)	Day 223 (62 days since last dose)	Lost to follow-up	Unknown	Unlikely related	Bupropion and lithium started on study day 223 (ongoing)/excluded due to a positive hepatitis/HIV screen	No relevant past history or concomitant medications

*UTI* urinary tract infection, *R* randomised, *SB* single blind, *DB* double blind, *PC* placebo controlled, *AC* active controlled, *O* open label, *PG* parallel group

^a^Family reported that subject had taken “poison.” The event was not assigned a body system and therefore does not appear in the pooled output for adverse events within the psychiatric disorders classification

^b^The sponsor considered chloroquine co-suspect

^c^Loading dose 200 mg/day for 3 days plus 200 mg weekly for 23 weeks

### Tafenoquine prophylaxis

Although this review focuses on tafenoquine for *P. vivax* relapse prevention, it is necessary to briefly discuss the prophylaxis studies as there are some features of these that should be explained in order to understand the safety signals for NPAEs at higher tafenoquine exposures.

In Table 5, 1445 patients received prophylaxis at the highest tafenoquine doses of > 300 mg > 3 days. However, 34.0% (492/1445) of subjects in the highest dose group were Australian Defence Force (ADF) personnel deployed on United Nations peacekeeping duties in East Timor, many of whom experienced a ‘war-like’ scenario [54]. A recent examination compared data for ADF subjects deployed in East Timor to non-ADF resident subjects who received the same tafenoquine regimen (200 mg loading dose for 3 days, then 200 mg weekly prophylaxis) [54]. In this analysis, all adverse events were more common in the deployed ADF group (467/492; 94.9%) than in resident non-ADF subjects (225/333; 67.6%), and psychiatric adverse events occurred in 5.1% (25/492) of ADF soldiers versus 2.1% (7/333) of resident non-ADF subjects [54, 79].

A possible explanation for this increased reporting of NPAEs in ADF personnel is that soldiers experienced traumatic events, including danger of being killed or injured (71%); seeing dead bodies (49%); fear of exposure to a toxic agent, contagious disease, or injury (31%); and having a friend/associate killed or injured (30%) [86]. Additionally, around 40% of soldiers who received tafenoquine sustained physical injuries as a result of their deployment while other illnesses, such as gastroenteritis and heat-related illnesses, were also common [54]. Thus, over the 6 months of tafenoquine administration, soldiers were exposed to many factors, other than tafenoquine, that could have contributed to their reporting of NPAEs and which confound causality assessment. Thus, the higher incidence of NPAEs at higher tafenoquine exposures not only reflects the long duration over which adverse events were recorded, but also the circumstances in which the drug was taken.

## Conclusions

There was no evidence of neurotoxicity, neurobehavioural disorder, or clinical neurotoxicity with tafenoquine at supra-therapeutic doses in rodent and non-rodent models [74–79]. These observations, along with the demonstration of minimal CNS penetration in the rat suggest that serious or severe CNS events are unlikely to occur with tafenoquine therapeutic doses in humans.

In *P. vivax* relapse prevention, with single-dose tafenoquine (300 mg)/chloroquine, no serious or severe CNS events were reported with the observed events being mild-to-moderate and self-limiting [9–11]. Tafenoquine/chloroquine did not increase the prevalence of NPAEs compared with placebo/chloroquine and the nature and frequency of NPAEs with tafenoquine/chloroquine were similar to that of primaquine/chloroquine [9–11]. Therefore, single-dose 300 mg tafenoquine/chloroquine for *P. vivax* malaria relapse prevention is anticipated to have a low risk of significant CNS effects [74]. However, it is recommended that patients disclose any prior mental health issues prior to tafenoquine prescribing.

In clinical trials, tafenoquine/chloroquine has been shown to reduce the risk of *P. vivax* relapse by approximately 70% over 6 months’ follow up compared with chloroquine alone, with a similar safety profile to primaquine [9–11, 87]. Single-dose tafenoquine offers complete adherence and presents an opportunity to expand *P. vivax* relapse prevention and improve clinical effectiveness over the current 14-day primaquine regimen [88]. Tafenoquine is now under evaluation with blood schizonticides other than chloroquine for relapse prevention in *P. vivax*.

Coupled with advances in rapid quantitative diagnostics for screening of patients for glucose-6-phosphate dehydrogenase deficiency, a contraindication for 8-aminoquinoline therapy, the risk:benefit profile for tafenoquine in *P. vivax* relapse prevention is favourable. The potential of tafenoquine for both reducing the burden of *P. vivax* malaria and draining the hypnozoite malaria transmission reservoir represents the first major advance in the treatment and control of *P. vivax* since primaquine was introduced nearly 70 years ago.

## Data Availability

All relevant data are provided in the manuscript or available from published materials as cited.

## Abbreviations

NPAE: Neuropsychological adverse events
ADF: Australian Defence Force

## References

[1] Lee SJ, Ter Kuile FO, Price RN, Luxemburger C, Nosten F (2017). Adverse effects of mefloquine for the treatment of uncomplicated malaria in Thailand: a pooled analysis of 19,850 individual patients. PLoS ONE.

[2] Eick-Cost AA, Hu Z, Rohrbeck P, Clark LL (2017). Neuropsychiatric outcomes after mefloquine exposure among US Military service members. Am J Trop Med Hyg.

[3] Ringqvist A, Bech P, Glenthoj B, Petersen E (2015). Acute and long-term psychiatric side effects of mefloquine: a follow-up on Danish adverse event reports. Travel Med Infect Dis.

[4] Toovey S (2009). Mefloquine neurotoxicity: a literature review. Travel Med Infect Dis.

[5] Bitta MA, Kariuki SM, Mwita C, Gwer S, Mwai L, Newton C (2017). Antimalarial drugs and the prevalence of mental and neurological manifestations: a systematic review and meta-analysis. Wellcome Open Res.

[6] Dow GS, Liu J, Lin G, Hetzell B, Thieling S, McCarthy WF (2015). Summary of anti-malarial prophylactic efficacy of tafenoquine from three placebo-controlled studies of residents of malaria-endemic countries. Malar J.

[7] Nasveld PE, Edstein MD, Reid M, Brennan L, Harris IE, Kitchener SJ (2010). Randomized, double-blind study of the safety, tolerability, and efficacy of tafenoquine versus mefloquine for malaria prophylaxis in nonimmune subjects. Antimicrob Agents Chemother.

[8] Schlagenhauf P, Hatz C, Behrens R, Visser L, Funk M, Holzer B (2015). Mefloquine at the crossroads? Implications for malaria chemoprophylaxis in Europe. Travel Med Infect Dis.

[9] Llanos-Cuentas A, Lacerda MV, Rueangweerayut R, Krudsood S, Gupta SK, Kochar SK (2014). Tafenoquine plus chloroquine for the treatment and relapse prevention of *Plasmodium vivax* malaria (DETECTIVE): a multicentre, double-blind, randomised, phase 2b dose-selection study. Lancet.

[10] Lacerda M, Llanos-Cuentas A, Krudsood S, Lon C, Saunders D, Mohammed R (2019). Single-dose tafenoquine to prevent relapse of *Plasmodium vivax* malaria. N Engl J Med.

[11] Llanos-Cuentas A, Lacerda M, Tinh Hien T, Vélez I, Namaik-larp C, Chu C (2019). Tafenoquine versus primaquine to prevent relapse of *Plasmodium vivax* malaria. N Engl J Med.

[12] Kim JR, Nandy A, Maji AK, Addy M, Dondorp AM, Day NP (2012). Genotyping of *Plasmodium vivax* reveals both short and long latency relapse patterns in Kolkata. PLoS ONE.

[13] Douglas NM, Poespoprodjo JR, Patriani D, Malloy MJ, Kenangalem E, Sugiarto P (2017). Unsupervised primaquine for the treatment of *Plasmodium vivax* malaria relapses in southern Papua: a hospital-based cohort study. PLoS Med.

[14] Chui CS, Chan EW, Wong AY, Root A, Douglas IJ, Wong IC (2016). Association between oral fluoroquinolones and seizures: a self-controlled case series study. Neurology.

[15] Bauquier SH, Jiang JL, Lai A, Cook MJ (2016). Clonic seizures in GAERS rats after oral administration of enrofloxacin. Comp Med.

[16] Sutter R, Ruegg S, Tschudin-Sutter S (2015). Seizures as adverse events of antibiotic drugs: a systematic review. Neurology.

[17] Dayan AD (1998). Neurotoxicity and artemisinin compounds: do the observations in animals justify limitation of clinical use?. Med Trop.

[18] Genovese RF, Newman DB (2008). Understanding artemisinin-induced brainstem neurotoxicity. Arch Toxicol.

[19] Kamchonwongpaisan S, McKeever P, Hossler P, Ziffer H, Meshnick SR (1997). Artemisinin neurotoxicity: neuropathology in rats and mechanistic studies in vitro. Am J Trop Med Hyg.

[20] Nontprasert A, Nosten-Bertrand M, Pukrittayakamee S, Vanijanonta S, Angus BJ, White NJ (1998). Assessment of the neurotoxicity of parenteral artemisinin derivatives in mice. Am J Trop Med Hyg.

[21] Nontprasert A, Pukrittayakamee S, Nosten-Bertrand M, Vanijanonta S, White NJ (2000). Studies of the neurotoxicity of oral artemisinin derivatives in mice. Am J Trop Med Hyg.

[22] Li Q, Hickman M (2011). Toxicokinetic and toxicodynamic (TK/TD) evaluation to determine and predict the neurotoxicity of artemisinins. Toxicology.

[23] Li QG, Mog SR, Si YZ, Kyle DE, Gettayacamin M, Milhous WK (2002). Neurotoxicity and efficacy of arteether related to its exposure times and exposure levels in rodents. Am J Trop Med Hyg.

[24] Efferth T, Kaina B (2010). Toxicity of the antimalarial artemisinin and its dervatives. Crit Rev Toxicol.

[25] Gordi T, Lepist EI (2004). Artemisinin derivatives: toxic for laboratory animals, safe for humans?. Toxicol Lett.

[26] Tiono AB, Tinto H, Alao MJ, Meremikwu M, Tshefu A, Ogutu B (2015). Increased systemic exposures of artemether and dihydroartemisinin in infants under 5 kg with uncomplicated *Plasmodium falciparum* malaria treated with artemether-lumefantrine (Coartem(R)). Malar J.

[27] Davis TM, Binh TQ, Ilett KF, Batty KT, Phuong HL, Chiswell GM (2003). Penetration of dihydroartemisinin into cerebrospinal fluid after administration of intravenous artesunate in severe falciparum malaria. Antimicrob Agents Chemother.

[28] Grabias B, Kumar S (2016). Adverse neuropsychiatric effects of antimalarial drugs. Expert Opin Drug Saf.

[29] Centers for Disease Control and Prevention. Malaria: disease. CDC. 2018. https://www.cdc.gov/malaria/about/disease.html. Accessed 24 Feb 2020.

[30] Bartoloni A, Zammarchi L (2012). Clinical aspects of uncomplicated and severe malaria. Mediterr J Hematol Infect Dis.

[31] Ssenkusu JM, Hodges JS, Opoka RO, Idro R, Shapiro E, John CC (2016). Long-term behavioral problems in children with severe malaria. Pediatrics.

[32] Boivin MJ, Bangirana P, Byarugaba J, Opoka RO, Idro R, Jurek AM (2007). Cognitive impairment after cerebral malaria in children: a prospective study. Pediatrics.

[33] John CC, Bangirana P, Byarugaba J, Opoka RO, Idro R, Jurek AM (2008). Cerebral malaria in children is associated with long-term cognitive impairment. Pediatrics.

[34] Fernando SD, Rodrigo C, Rajapakse S (2010). The ‘hidden’ burden of malaria: cognitive impairment following infection. Malar J.

[35] Zimmerman GA, Castro-Faria-Neto H (2010). Persistent cognitive impairment after cerebral malaria: models, mechanisms and adjunctive therapies. Expert Rev Anti Infect Ther..

[36] Bangirana P, Menk J, John CC, Boivin MJ, Hodges JS (2013). The association between cognition and academic performance in Ugandan children surviving malaria with neurological involvement. PLoS ONE.

[37] Bangirana P, Musisi S, Boivin MJ, Ehnvall A, John CC, Bergemann TL (2011). Malaria with neurological involvement in Ugandan children: effect on cognitive ability, academic achievement and behaviour. Malar J.

[38] Holding PA, Stevenson J, Peshu N, Marsh K (1999). Cognitive sequelae of severe malaria with impaired consciousness. Trans R Soc Trop Med Hyg.

[39] Idro R, Kakooza-Mwesige A, Asea B, Ssebyala K, Bangirana P, Opoka RO (2016). Cerebral malaria is associated with long-term mental health disorders: a cross sectional survey of a long-term cohort. Malar J.

[40] Jones R, Kunsman G, Levine B, Smith M, Stahl C (1994). Mefloquine distribution in postmortem cases. Forensic Sci Int.

[41] Pham YT, Nosten F, Farinotti R, White NJ, Gimenez F (1999). Cerebral uptake of mefloquine enantiomers in fatal cerebral malaria. Int J Clin Pharmacol Ther.

[42] Dow G, Bauman R, Caridha D, Cabezas M, Du F, Gomez-Lobo R (2006). Mefloquine induces dose-related neurological effects in a rat model. Antimicrob Agents Chemother.

[43] Dow GS, Hudson TH, Vahey M, Koenig ML (2003). The acute neurotoxicity of mefloquine may be mediated through a disruption of calcium homeostasis and ER function in vitro. Malar J.

[44] Toovey S, Bustamante LY, Uhlemann AC, East JM, Krishna S (2008). Effect of artemisinins and amino alcohol partner antimalarials on mammalian sarcoendoplasmic reticulum calcium adenosine triphosphatase activity. Basic Clin Pharmacol Toxicol.

[45] Viel JF, Warembourg C, Le Maner-Idrissi G, Lacroix A, Limon G, Rouget F (2015). Pyrethroid insecticide exposure and cognitive developmental disabilities in children: the PELAGIE mother-child cohort. Environ Int.

[46] Wnuk A, Rzemieniec J, Litwa E, Lason W, Krzeptowski W, Wojtowicz AK (2016). The crucial involvement of retinoid X receptors in DDE neurotoxicity. Neurotox Res.

[47] Soderlund DM (2012). Molecular mechanisms of pyrethroid insecticide neurotoxicity: recent advances. Arch Toxicol.

[48] Yatham S, Sivathasan S, Yoon R, da Silva TL, Ravindran AV (2017). Depression, anxiety, and post-traumatic stress disorder among youth in low and middle income countries: a review of prevalence and treatment interventions. Asian J Psychiatr.

[49] Gomez-Restrepo C, Cruz-Ramirez V, Medina-Rico M, Rincon CJ (2018). Mental health in displaced children by armed conflict—National Mental Health Survey Colombia 2015. Actas Esp Psiquiatr.

[50] World Bank. Malaria. World Bank. 2015. http://www.worldbank.org/en/topic/health/brief/malaria. Accessed 24 Feb 2020.

[51] Salgado-Delgado R, Tapia Osorio A, Saderi N, Escobar C (2011). Disruption of circadian rhythms: a crucial factor in the etiology of depression. Depress Res Treat.

[52] Kim JN, Lee BM (2018). Risk management of free radicals involved in air travel syndromes by antioxidants. J Toxicol Environ Health B Crit Rev.

[53] Rundle AG, Revenson TA, Friedman M (2018). Business travel and behavioral and mental health. J Occup Environ Med.

[54] Novitt-Moreno A, Ransom J, Dow G, Smith B, Read LT, Toovey S (2017). Tafenoquine for malaria prophylaxis in adults: an integrated safety analysis. Travel Med Infect Dis.

[55] Kessler RC, Merikangas KR (2004). The National Comorbidity Survey Replication (NCS-R): background and aims. Int J Methods Psychiatr Res.

[56] Kessler RC, Heeringa SG, Stein MB, Colpe LJ, Fullerton CS, Hwang I (2014). Thirty-day prevalence of DSM-IV mental disorders among nondeployed soldiers in the US Army: results from the Army Study to Assess Risk and Resilience in Servicemembers (Army STARRS). JAMA Psychiatry.

[57] Schoenbaum M, Kessler RC, Gilman SE, Colpe LJ, Heeringa SG, Stein MB (2014). Predictors of suicide and accident death in the Army Study to Assess Risk and Resilience in Servicemembers (Army STARRS): results from the Army Study to Assess Risk and Resilience in Servicemembers (Army STARRS). JAMA Psychiatry.

[58] McFarlane A, Hodson S, N VH, C D. Mental health in the Australian Defence Force: 2010 ADF mental health prevalence and wellbeing study report. Department of Defence. 2011. http://www.defence.gov.au/health/dmh/docs/mhpwsreport-fullreport.pdf. Accessed 24 Feb 2020.

[59] Rusu C, Zamorski MA, Boulos D, Garber BG (2016). Prevalence comparison of past-year mental disorders and suicidal behaviours in the Canadian Armed Forces and the Canadian general population. Can J Psychiatry.

[60] Goodwin L, Wessely S, Hotopf M, Jones M, Greenberg N, Rona RJ (2015). Are common mental disorders more prevalent in the UK serving military compared to the general working population?. Psychol Med.

[61] Nock MK, Stein MB, Heeringa SG, Ursano RJ, Colpe LJ, Fullerton CS (2014). Prevalence and correlates of suicidal behavior among soldiers: results from the Army Study to Assess Risk and Resilience in Servicemembers (Army STARRS). JAMA Psychiatry.

[62] Friedman MJ (2014). Suicide risk among soldiers: early findings from Army Study to Assess Risk and Resilience in Servicemembers (Army STARRS). JAMA Psychiatry.

[63] Mulligan K, Jones N, Davies M, McAllister P, Fear NT, Wessely S (2012). Effects of home on the mental health of British forces serving in Iraq and Afghanistan. Br J Psychiatry.

[64] Trautmann S, Goodwin L, Hofler M, Jacobi F, Strehle J, Zimmermann P (2017). Prevalence and severity of mental disorders in military personnel: a standardised comparison with civilians. Epidemiol Psychiatr Sci.

[65] Ursano RJ, Stein MB, Herberman Mash HB, Naifeh JA, Fullerton CS, Zaslavsky AM (2018). Documented family violence and risk of suicide attempt among US Army soldiers. Psychiatry Res.

[66] Ministry of Defence. UK Armed Forces mental health: annual summary & trends over time, 2007/08–2016/17. MOD. 2017. www.gov.uk/government/statistics/mental-health-in-the-uk-armed-forces-background-quality-report. Accessed 24 Feb 2020.

[67] Kim HM, Levine DS, Pfeiffer PN, Blow AJ, Marchiondo C, Walters H (2017). Postdeployment suicide risk increases over a 6-month period: predictors of increased risk among midwestern Army National Guard soldiers. Suicide Life Threat Behav.

[68] Lazar SG (2014). The mental health needs of military service members and veterans. Psychodyn Psychiatry.

[69] Hines LA, Sundin J, Rona RJ, Wessely S, Fear NT (2014). Posttraumatic stress disorder post Iraq and Afghanistan: prevalence among military subgroups. Can J Psychiatry.

[70] Hoge CW, Castro CA, Messer SC, McGurk D, Cotting DI, Koffman RL. Combat duty in Iraq and Afghanistan, mental health problems and barriers to care. US Army Med Dep J. 2008;7–17. 20088060

[71] Sareen J, Cox BJ, Afifi TO, Stein MB, Belik SL, Meadows G (2007). Combat and peacekeeping operations in relation to prevalence of mental disorders and perceived need for mental health care: findings from a large representative sample of military personnel. Arch Gen Psychiatry.

[72] Adshead S (2014). The adverse effects of mefloquine in deployed military personnel. J R Nav Med Serv.

[73] Schmidt LH (1983). Relationships between chemical structures of 8-aminoquinolines and their capacities for radical cure of infections with *Plasmodium cynomolgi* in Rhesus monkeys. Antimicrob Agents Chemother.

[74] GlaxoSmithKline. Krintafel (tafenoquine succinate tablets): FDA Advisory Committee Briefing Document GSK. 2018. https://www.fda.gov/downloads/advisorycommittees/committeesmeetingmaterials/drugs/anti-infectivedrugsadvisorycommittee/ucm612875.pdf. Accessed 24 Feb 2020.

[75] Dow GS, Brown T, Reid M, Smith B, Toovey S (2017). Tafenoquine is not neurotoxic following supertherapeutic dosing in rats. Travel Med Infect Dis.

[76] Dow GS, Gettayacamin M, Hansukjariya P, Imerbsin R, Komcharoen S, Sattabongkot J (2011). Radical curative efficacy of tafenoquine combination regimens in *Plasmodium cynomolgi*-infected Rhesus monkeys (*Macaca mulatta*). Malar J.

[77] Puri SK, Dutta GP (2003). Blood schizontocidal activity of WR 238605 (tafenoquine) against *Plasmodium cynomolgi* and *Plasmodium fragil*e infections in Rhesus monkeys. Acta Trop.

[78] DiTusa C, Kozar MP, Pybus B, Sousa J, Berman J, Gettayacamin M (2014). Causal prophylactic efficacy of primaquine, tafenoquine, and atovaquone-proguanil against *Plasmodium cynomolgi* in a rhesus monkey model. J Parasitol.

[79] Sixty Degrees Pharmaceuticals. ARAKODA™ (tafenoquine succinate) tablets for the prevention of malaria in adults: NDA 210607; briefing document for the Antimicrobial Drugs Advisory Committee. Sixty Degrees Pharmaceuticals. 2018. https://www.fda.gov/downloads/AdvisoryCommittees/CommitteesMeetingMaterials/Drugs/Anti-InfectiveDrugsAdvisoryCommittee/UCM614202.pdf. Accessed 24 Feb 2020.

[80] Fukuda MM, Krudsood S, Mohamed K, Green JA, Warrasak S, Noedl H (2017). A randomized, double-blind, active-control trial to evaluate the efficacy and safety of a three day course of tafenoquine monotherapy for the treatment of *Plasmodium vivax* malaria. PLoS ONE.

[81] Walsh DS, Wilairatana P, Tang DB, Heppner DG, Brewer TG, Krudsood S (2004). Randomized trial of 3-dose regimens of tafenoquine (WR238605) versus low-dose primaquine for preventing *Plasmodium vivax* malaria relapse. Clin Infect Dis.

[82] Schmidt IG, Schmidt LH (1951). Neurotoxicity of the 8-aminoquinolines. III. The effects of pentaquine, isopentaquine, primaquine, and pamaquine on the central nervous system of the Rhesus monkey. J Neuropathol Exp Neurol.

[83] Berman J, Brown T, Dow G, Toovey S (2018). Tafenoquine and primaquine do not exhibit clinical neurologic signs associated with central nervous system lesions in the same manner as earlier 8-aminoquinolines. Malar J.

[84] Sixty Degrees Pharmaceuticals. ARAKODA™ (tafenoquine) tablets, for oral use; prescribing information. Sixty Degrees Pharmaceuticals. 2018. https://www.accessdata.fda.gov/drugsatfda_docs/label/2018/210607lbl.pdf. Accessed 24 Feb 2020.

[85] GlaxoSmithKline. KRINTAFEL (tafenoquine) tablets, for oral use; prescribing information. GlaxoSmithKline. 2018. https://www.accessdata.fda.gov/drugsatfda_docs/label/2018/210795s000lbl.pdf. Accessed 24 Feb 2020.

[86] Waller M, Treloar SA, Sim MR, McFarlane AC, McGuire AC, Bleier J (2012). Traumatic events, other operational stressors and physical and mental health reported by Australian Defence Force personnel following peacekeeping and war-like deployments. BMC Psychiatry.

[87] Rueangweerayut R, Bancone G, Harrell EJ, Beelen AP, Kongpatanakul S, Mohrle JJ (2017). Hemolytic potential of tafenoquine in female volunteers heterozygous for glucose-6-phosphate dehydrogenase (G6PD) deficiency (G6PD Mahidol variant) versus G6PD-normal volunteers. Am J Trop Med Hyg.

[88] Galappaththy GN, Tharyan P, Kirubakaran R (2013). Primaquine for preventing relapse in people with *Plasmodium vivax* malaria treated with chloroquine. Cochrane Database Syst Rev.

[89] Shanks GD, Oloo AJ, Aleman GM, Ohrt C, Klotz FW, Braitman D (2001). A new primaquine analogue, tafenoquine (WR 238605), for prophylaxis against *Plasmodium falciparum* malaria. Clin Infect Dis.

[90] Brueckner RP, Lasseter KC, Lin ET, Schuster BG (1998). First-time-in-humans safety and pharmacokinetics of WR 238605, a new antimalarial. Am J Trop Med Hyg.

[91] Green JA, Patel AK, Patel BR, Hussaini A, Harrell EJ, McDonald MJ (2014). Tafenoquine at therapeutic concentrations does not prolong Fridericia-corrected QT interval in healthy subjects. J Clin Pharmacol.

[92] Leary KJ, Riel MA, Roy MJ, Cantilena LR, Bi D, Brater DC (2009). A randomized, double-blind, safety and tolerability study to assess the ophthalmic and renal effects of tafenoquine 200 mg weekly versus placebo for 6 months in healthy volunteers. Am J Trop Med Hyg.

